# Thermotropic and Thermochromic Polymer Based Materials for Adaptive Solar Control

**DOI:** 10.3390/ma3125143

**Published:** 2010-12-06

**Authors:** Arno Seeboth, Ralf Ruhmann, Olaf Mühling

**Affiliations:** Department Chromogenic Polymers, Fraunhofer Institute for Applied Polymer Research, Volmerstrasse 7B, D-12489 Berlin, Germany; E-Mails: ralf.ruhmann@iap.fraunhofer.de (R.R.); olaf.muehling@iap.fraunhofer.de (O.M.)

**Keywords:** thermotropic, thermochromic, polymer, sun protection, solar control

## Abstract

The aim of this review is to present the actual status of development in adaptive solar control by use of thermotropic and organic thermochromic materials. Such materials are suitable for application in smart windows. In detail polymer blends, hydrogels, resins, and thermoplastic films with a reversible temperature-dependent switching behavior are described. A comparative evaluation of the concepts for these energy efficient materials is given as well. Furthermore, the change of strategy from ordinary shadow systems to intrinsic solar energy reflection materials based on phase transition components and a first remark about their realization is reported. Own current results concerning extruded films and high thermally stable casting resins with thermotropic properties make a significant contribution to this field.

## 1. Introduction

Chromogenic materials are perfectly suited for application in solar control glazing [1,2,3]. The motivation for scientists and engineers to develop novel electrochromic, thermochromic and thermotropic materials is their potential technical application. Usually, conventional non-switchable sun protection films combine reflection and absorbance of the solar infrared and UV radiation and lead to a noticeable reduction of heat during the summer months [4]. In contrast, thermochromic and thermotropic systems can be compared regarding their influence on the energy efficiency of the buildings. Here, the thermotropic materials, acting on the basis of reflective effects, seem to be more advantageous. The reflection partially prevents solar irradiation from entering the building as well as providing the windows or façade elements and is therefore favored in comparison to thermochromic systems. Thermochromic systems stand out for applications where a permanent view from the inside out is needed.

The dynamic control of daylight has been termed the “Holy Grail of the Fenestration Industry” [5]. To that effect, the R&D effort in this field is directed on a glazing that will dynamically and reversibly modulate its total solar and/or visible transmittance properties [5]. The adaptation of the optical properties of the glazing or façade elements to the outer conditions can be triggered by passive outer stimuli like temperature or light intensity itself or by an active electrically controlled switching. After several years of intensive work, different promising technologies are entering the market of day-lighting applications.

At this stage, electrochromic smart windows appear to be the technology with the highest degree of commercialization and are offered by some companies. Electrochromic coatings are multilayer thin films that are actively controlled by a voltage, and switch reversibly from a clear state to a dark state. The transmittance changes are linked with changes in solar heat gain coefficient (SHGC) or G-Value (G-value is the coefficient commonly used in Europe and SHGC in the United States).

In comparison to actively switching systems, the advantages of passively switching systems in roofs, façade elements, sun shades and overhead lights comprise the following aspects:

- No additional energy consumption, no electrical power supply
- High switching range between off- and on-state
- Variety in shape and size—three-dimensional convex glasses
- Low cost product and low effort for installation as well
- Self-regulating, *i.e*., no active control is required for operation
- High long-term stability, maintenance-free
- High potential to replace conventional sun protection systems like sun-blinds, roller blinds, *etc*.

The first four items represent unique features. In particular, it is amazing that the solar energy itself is used as a promoter against sun heat.

Therewith, the passively switching smart windows are an interesting field of investigation directed at enhancing the energy efficiency of buildings. The review presented here is focused on the application of passively switching, polymer based materials for adaptive sun protection in buildings.

The review is structured as follows. After starting with theoretical considerations, thermotropic systems like polymer blends, hydrogels, casting resins and thermoplastic films are described in more detail. Then, thermochromic phenomena in polymers [6] are discussed. New approaches to exploit nanoparticles in a polymeric matrix for sun protection glazing are also included. Finally, the developments setting the future trends in the field of the so-called “smart windows” will be examined. 

## 2. Thermotropic Systems

Thermotropic systems exhibit a temperature dependent change of the light scattering properties. If the increased scattering is linked with a substantial degree of back scattering dependent on the temperature increase, the materials are suited for an application in solar control. Thermotropic effects investigated in this field can be caused by a phase separation process, by a phase transition between an isotropic and an anisotropic (liquid-crystalline) state and by strongly differing temperature dependencies of the refractive indices of domains and matrix. Polymer blends and polymeric hydrogels have been studied intensively.

### 2.1. Theoretical Considerations

The most active work in creating a passively switching sun protection glazing has been based on thermotropic materials. These systems consist of at least two components with different refractive indices (RI). At temperatures below the switching point, the difference in refractive index has no effect because all components are homogeneously mixed at a molecular level or have similar RIs. In this state, the material has a median refractive index and is highly transparent for solar radiation. Due to a phase separation or phase transition upon reaching the switching temperature, a difference between the refractive indices of both components appears. As a consequence, scattering domains of dimensions similar to solar wavelengths take effect. The solar radiation is scattered at the interface between both components. The incident solar radiation is reflected (back-scattered).

Some papers deal with an ideal configuration and simulate the scattering process to assess the influence of different parameters like domain size, refractive index difference and volume fraction of scattering domains [7,8]. Nitz calculations are based on the Lorenz-Mie theory [9], which is an exact analytical solution of the Maxwell equations for the scattering of electromagnetic radiation by spherical domains. In principle, this theory is applicable to spheres of all sizes, refractive indices, and for radiation at all wavelengths. For domain sizes much smaller or larger than wavelength approximations can be used, namely Rayleigh scattering and geometrical optics, respectively [10].

For discrete spherical particles embedded in a transparent matrix, the reduction of transmitted light intensity *I*/*I*_0_ as a result of scattering can be described by Equation 1, in which *V*_SD_ is the volume fraction of the scattering domains (SD), *x* the optical path-length, *r*_SD_ the SD radius, *λ* the wavelength of light, and *n*_SD_ and *n*_M_ refractive indices (RI) of the scattering domains and matrix, respectively.

(1) $$\frac{I}{I0}\sim \;exp\left[\frac{-3V_{SD}x{r_{SD}}^{3}}{4\lambda^{4}}\left(\frac{n_{SD}}{n_{M}}-1\right)\right]$$

Equation 1 clarifies that for a given wavelength and in the absence of RI matching (*n*_SD_/*n*_M_ ≠ 1), the transmitted light intensity can be reduced particularly by increasing the SD radius (*r*_SD_^3^). The influence of the remaining parameters (volume fraction of the SDs, optical path length, RI ratio) is significantly weaker. This can be impressively demonstrated when the SD size is about 1–2 orders of magnitude smaller than the wavelength of light. In this case, a domain/matrix-composite looks to light like a continuous medium and scattering is minimized.

In view of the best possible solar light shielding properties, a high back scattering (reflection) efficiency is required. However, equation 1 does not consider the angular distribution of the scattered light. In order to distinguish between forward and back scattering the utilization of the Lorenz-Mie theory is necessary. In the case of Rayleigh scattering (*r*_SD_ << *λ*), the angular distribution is symmetric implying that forward and back scattering are equally probable (amount of forward scattering = amount of back scattering = 0.5). However, in this domain size region, the total scattering efficiency is several magnitudes of order lower (compared to *r*_SD_ ≈ *λ*) but rises very steeply with increasing domain size. If the SD size reaches the wavelength region of light, the angular distribution of scattering changes radically and, thus, the Rayleigh approximation loses its validity. While the total scattering efficiency with increasing SD size still rises, more and more light is scattered into the forward hemisphere, *i.e*., the amount of back scattering is reduced considerably compared to Rayleigh scattering (<< 0.5). As a result, the back scattering efficiency (= total scattering efficiency • amount of back scattering) shows a more or less distinct maximum at a special SD size representing the optimum back scattering for the selected wavelength or for an appropriate spectral distribution. 

Nitz has found that for an efficient back scattering of solar radiation, the mean diameter *d* of the SDs should be in the range of *d* ≈ 200–400 nm. For *d* < 200 nm, the back-scattering efficiency decreases significantly. However, towards larger domain sizes (*d* ≈ 400–1,000 nm) the optimum width is relatively broad. For this reason, only small variations in optical properties can be expected for SD diameters between approximately 200–1000 nm. Additionally, it was observed that the position of the back scattering maximum with respect to the domain size is hardly affected by the refractive indices of matrix and scattering domain. In spite of large differences in their refractive indices, polymer blends (*n*_SD_ = 1.447, *n*_M_ = 1.529) and hydrogels (*n*_SD_ = 1.552, *n*_M_ = 1.343) offer similar results. Comparable results can be expected for thermotropic systems based on solid-liquid phase transitions of long-chain compounds.

### 2.2. Switching Mechanisms

Four different switching mechanisms have been reported for thermotropic systems:

- Phase separation (e.g., polymer blends and polymer gels with “Lower Critical Solution Temperature”. In the context of gel-based smart windows, the term Lower Critical Solution Temperature is often used. However, chemically crosslinked gels are not dissolved at any time, and thus, some authors in the more scientifically affected literature call this temperature for gels the “Volume Transition Temperature”);
- Change of the particle size (e.g., thermotropic nanoparticles);
- Aggregation (e.g., block copolymers);
- Phase transition (e.g., casting resins with fixed domains).

Polymer solutions, such as binary polymer blends or polymer gels, often show miscibility gaps. The appearance of these gaps is strongly influenced by the temperature. For an application in sun protection, material solutions with a lower critical solution temperature (LCST) are of special interest. Below the LCST, the blend is a homogeneously distributed mixture of both polymers with a uniform refractive index. Above the LCST, both polymers separate and one polymer forms domains in the matrix of the other polymer. If the refractive indices of the polymers are different, light scattering appears.

In a special assembly, the hydrogel with LCST forms the shell of a polymer nanoparticle. Heating above the LCST leads here to a deswelling of the shell and therewith to a drastic reduction of the particle size as well as to a drastic increase of the refractive index. The occurring refractive index difference between the particles and the surrounding water affects the formation of scattering domains and the back-scattering of the incident solar radiation.

An opposite approach employs the formation of micelles and clusters from the solution of block copolymers with increasing temperature. The size of the clusters enable their use as scattering domains in the surrounding water and the back-scattering of the incident solar radiation can be achieved.

A further concept employs temperature-induced phase transitions for the thermotropic behavior in sun protection applications [11]. The systems consist of at least two permanent main components. A thermotropic additive (domain material) is homogeneously dispersed in an appropriate matrix material. In contrast to systems based on a phase separation, the domains are fixed inside the matrix and they exist at every temperature. Below the switching temperature, the refractive indices of domain and matrix are (almost) equal and scattering is minimized. At the switching temperature, a phase transition proceeds in the domain material and its refractive index changes abruptly. So, in the on-state, the fixed domains are working as scattering centers affecting a partial reflection of the incident solar radiation. Examples of this concept are demonstrated in casting resins for laminated glazing [12,13,14,15] and in polyolefine films [16].

### 2.3. Phase-Separating Polymer Blends

Phase-separating polymer blends consist of a thermoplastic component embedded in a crosslinked matrix (semi-interpenetrating networks). The crosslinking of the matrix polymer ensures the reversibility of the phase separation and the mechanical stability of the composed layer. Furthermore, the network limits the maximal size of the domains of the thermoplastic component and leads to a high degree of backscattering of the incident sun radiation in the on-state. Also, the variation of the network density directly influences the resulting switching temperature of the thermotropic blend.

Thermotropic polymer blends composed of a chlorinated rubber and polymethacrylates were first described in 1993 by Röhm [17]. The switching temperature denoting the change from a transparent to a translucent state could be varied in the range from 60 °C to 140 °C. Thermotropic polymer blends with a switching temperature between 30 °C and 40 °C better suited for application in sun protection and a high reversibility of the switching process were also presented in 1993 [18]. The material consists of a poly(propylene oxide) and a styrene-hydroxyethyl methacrylate copolymer thermally crosslinked with a trifunctional isocyanate. In particular, crosslinking to a semi-interpenetrating network enhances the phase separation above the switching temperature and the reversibility of the separation process. A further improvement could be achieved by a photo-initiated polymerization and crosslinking process, enabling films with more uniform optical quality [19]. Thick films of 400 µm of the thermally crosslinked polymer blend exhibited a change of the normal-hemispherical transmittance from 92% to 30% at 20 °C and 90 °C, respectively [20]. Comparable results could be achieved with the UV crosslinked polymer blend. A 600 µm thick film changed its normal-hemispherical transmittance from 89% at 30 °C to 38% at 85 °C [21].

In spite of an investigation into thermotropic polymer blends for several years [22], due to technological problems, the development of smart windows based on phase separating polymer blends was interrupted and no products were commercialized [23].

### 2.4. Phase-Separating Thermotropic Gels

Thermotropic polymer gels for sun protection have been studied very extensively for several years. Already, the first prototype of a sun protection glazing based on a thermotropic polymer gel was worked into the residence of the Munich Zoo over a period of about 10 years from 1950.

Thermotropic polymer gels are formed from synthetic or natural polymers with varying amounts of water or organic solvents. Their application utilizes a temperature dependent transmittance change caused by phase separation processes. 

Furthermore, in this context, transmittance changes of hydrogels caused by lyotropic liquid-crystalline phases or by temperature-dependent micelle formation were investigated. Polymers like hydroxypropyl cellulose, poly(benzyl glutamate) and poly(vinyl alcohol) were intensively studied due to their high solubility in water and their pronounced lyotropic liquid-crystalline behavior [24]. For this purpose, solutions of an epoxylated poly(vinyl alcohol) or of mixtures of poly(ethylene glycol) and poly(vinyl alcohol) were crosslinked with borax. These hydrogels were found to possess thermotropic properties within wide concentration ranges. Unfortunately, on heatin, g the optical appearance of these kind of hydrogels changed from opaque to a transparent state. An application in sun protection failed.

Gels prepared from poly(N-isopropyl acrylamide) homo- and copolymers were extensively studied because of their high potential in different application fields [25]. They offer better opportunities than the lyotropic gel systems [26,27,28], and consequently, poly(N-isopropyl acryl amide) (PNIPAAm) is the first reported non-ionic hydrogel exhibiting a temperature dependent phase transition [29]. On heating to about 34 °C, it changes reversibly from a gel state into a collapsed state [30]. The switching temperature can be influenced either by copolymerization or by variation of the water content of the swelling agent. The investigation was handicapped by the strong volume reduction during phase transition. Therefore, only non-sealed glass-gel-glass sandwich constructions could be prepared. A 1 mm thick gel layer exhibited an excellent normal-normal transmittance change from about 95% at room temperature to less than 1% above the switching temperature. However, the appearance of a surface pattern caused by the strong volume reduction of the gel prevented a commercial application.

A first attempt of commercialization was done with Cloud Gel™ offered by Suntek since 1995 [31,32]. Here, the thermotropic gel is based on poly(methyl vinyl ether) crosslinked with methylene-bis-acrylamide. By varying the composition of the gel, the switching temperature can be adjusted between 10 °C and 65 °C [33]. The visible normal-hemispherical transmittance of a 1 mm thick layer changes from 92% to 6% at 25 °C and 50 °C, respectively. In addition, a high contrast ratio, *i.e*., a very steep transmittance change, could be detected. Immediately above the transition temperature, the transmittance dropped to about 65%. In comparison to polymer blends, Cloud Gel™ exhibits larger and very steep transmittance changes, recommending them for sun protection applications.

Besides chemically crosslinked gels, thermotropic gels with a physical network have been developed [34,35,36]. This type of thermotropic gels consist of polyalkoxides with ethyleneoxide and propyleneoxide monomer units and salts or other complex forming compounds as crosslinker. The ratio between both monomeric units as well as the crosslinking agent can be varied and, therewith, it is possible to adjust the switching temperature between the clear and the opaque, light scattering state in the range from room temperature to 80 °C. The optical performance of these thermotropic gels is similar to that of Cloud Gel™. However, advantageously, physically crosslinked gels keep a constant volume during the switching process. The applicability in smart windows is enhanced substantially [37].

Due to their biodegradability and commercial availability at low costs, biopolymers are promising in the field of thermotropic materials [38]. Depending on the composition the crosslinked hydrogels made from cellulose, amphiphiles and sodium chloride switching temperature between room temperature and 60 °C can be achieved. Here, the amphiphiles prevent the irreversible flocation of the cellulose derivatives. A 1 m^2^ large assembly of a sun protection glazing was shown to function fine over a period of two years under practical conditions. Named “Affinity Intelligent Window”, the product was marketed by its inventor [39]. Furthermore, thermotropic hydrogels based on cellulose derivatives, even without the addition of amphiphiles, are reported, with a satisfying switching behavior and with excellent long-term stability [40].

Further materials, e.g., hydrogels based on poly(ethylenglycol) macromonomers or on polymers from dimethylaminoethylmethacrylate (DMEMA) exhibit a temperature induced transmittance change, but their application in smart windows has not been investigated up to now [41,42].

A smart window assembly equipped with additional heating by indium tin oxide electrodes (ITO) is described too. A combination of active and passive switching is enabled and was investigated in more detail [43,44]. These hybrid light filters can be switched by solar as well as by electrical energy. The optical properties as well as energy consumption and temperature changes during the switching process have been studied. Besides application for the optical and thermal control in greenhouses and water boiling systems, it was concluded that electrically adjustable gel glasses can be used additionally as optically switched room partitions in indoor applications and also as novel material for large displays with low operational and prime costs. Nevertheless, a further significant reduction of the wattage is required by optimizing the outer components of the glazing. Furthermore, a thermotropic hydrogel with a low heat capacity is needed.

It should be noted that no electrolysis of the water based gels was observed. This means that no free water exists in the gel [43].

Thermotropic gels possess excellent optical properties, they are cheap and light stable. However, until now, no technology could be developed to produce an economically-sufficient surface area in time.

### 2.5. Thermotropic Nanoparticles and Aggregates

A recent approach to utilize polymeric hydrogels includes nanoparticles [45]. The thermotropic effect was caused by a variation of the particle size, depending on the temperature. For this, water-dispersed thermotropic nanoparticles with core-shell structures were synthesized by the *in situ* polymerization of a lightly crosslinked shell of PNIPAAm onto blue polystyrene cores. At room temperature, the thermal responsive outer shell is hydrophilic and is in a fully swollen gel state; but as the temperature rises above 31 °C, it becomes increasingly hydrophobic and eventually collapses as the temperature reaches the LCST of the PNIPAAm. Passing the LCST has a drastic effect on the color of the latex solution, which exhibits an intense blue color at room temperature and gradually pales or lightens as the temperature rises above 31 °C. This is a reversible process. Simultaneously, above the LCST, the hydrogel shrinks dramatically leading to a reduction of the particle size from about 1,000 to 430 nm. As a consequence, the refractive index of the nanoparticles and the difference to the surrounding matrix are strongly increased. Therewith, above the LCST, an increase of the scattering efficiency of the system can be observed. The normal-normal transmittance at 630 nm decreases from 58% at 20 °C to 10% at 42 °C. The weakly scattering, almost clear system becomes opaque; thus, its properties should be tested in more detailed for sun protection glazing.

**Figure 1:**
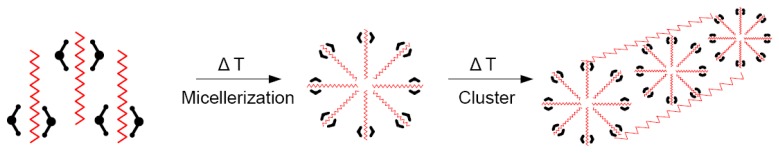
Cluster formation of EPE molecules dispersed in water.

A further approach employs the aggregation of a triblock copolymer dispersed in water to establish the design of a novel prototype smart window [46]. The thermo reversible clear-opaque switching is based on the thermally induced aggregation of a poly(ethylene oxide-co-propylene oxide-co-ethylene oxide) triblock terpolymer (EPE) [47]. With increasing temperature, the homogeneously dispersed EPE molecules firstly form micelles. Further heating leads to the packing of the micelles into clusters (Figure 1).

A 1 mm thick layer of water containing 1 vol. % of EPE exhibits a normal-normal transmittance change from about 85% to nearly 0% at 600 nm.

The addition of sodium dodecyl sulfate (SDS) influences the formation of the micelles and clusters. Therewith, the switching temperature can be tuned in the range from 25 °C to about 60 °C by mixing an aqueous solution of EPE with varying amounts of the surfactant SDS. Furthermore, the authors described a smart window assembly equipped with an additional heating by ITO electrodes; an active switching is enabled.

Without any question, both strategies—nanoparticles and aggregates as well—could be helpful suggestions to develop the topic. However, systems without a constant thickness layer or any aqueous medium with free water, such as the case using SDS micelles, in combination with electrode application appear to be more of an academic interest at time.

### 2.6. Polymer Blends with Phase Transition

The attention is now turned to blends composed of two permanently immiscible polymers with strongly differing temperature dependencies of their refractive indices. In the off-state, the refractive indices of both polymers are almost equal and the polymer film appears transparent. With increasing temperature, a difference between the refractive indices of both polymers arises and light scattering appears, e.g., blends made from transparent polyamides (PA) as matrix polymer with domain forming copolymers from ethylene and glycidyl methacrylate (E-co-GMA) are suggested [48]. The temperature dependent optical behavior of such polymer blends, varying the content of the matrix polymer from 90% to 100%, is summarized in Table 1.

**Table 1:** Normal-normal transmittance data of PA/P(E-co-GMA) polymer blends [48] with a thickness of 4 mm.

Sample	Composition [%]		Normal-normal transmittance at 560 nm [%]					
	PA	P(E-co-GMA)	30 °C	40 °C	50 °C	60 °C	70 °C	80 °C
1	100	0	85	85	85	85	85	85
2A	98	2	77	75	70	60	50	45
2B	95	5	65	60	55	40	30	25
2C	91	9	45	35	20	8	2	0

As can be clearly seen, the performance properties of this material type are characterized by a broad transition temperature range from 35 °C to 95 °C. A steep transition from the clear to the opaque state within a small temperature range is not detected, indicating that only the glass transition of the domain polymer is involved in the transmittance change. Probably, the refractive index of the domain polymer depends strongly on the temperature when above its glass transition temperature, whereas the matrix polymer exhibits only a moderate dependency of its refractive index on temperature when below its glass transition temperature. Thus, the large difference of the refractive indices of the involved polymers causes the opacity at elevated temperatures.

Nevertheless, a 2 mm thick thermotropic layer showed a normal-hemispherical solar transmittance of 82% in the clear state (25 °C) and 57% in the opaque state (95 °C) [49]. Based on these promising results, further development directed at adaptation of the switching temperature and efficiency can provide materials with suitability for sun protection. Recent results support this concept [50].

### 2.7. Thermotropic Casting Resins

Phase transition systems for solar control glazing have been almost exclusively described in the patent literature [51,52,53,54,55,56,57]. Generally, long-chain compounds with a solid-liquid phase transition in the desired temperature range (e.g., alkanes, fatty acid esters) are used as domain materials and mixed with casting resins used in laminated glasses. Various switching temperatures within the range of 10 °C to 50 °C can be realized.

Based on the results of the Fraunhofer Gesellschaft [58], the sun protecting glazing T-Opal^®^ consisting of a double float glazing with thermotropic casting resin in between was introduced. The thermotropic casting resin is composed of a UV curing matrix polymer and an (partially) immiscible low molecular weight additive. The additive forms a separate phase in the cured matrix polymer and exhibits a discontinuous change of the refractive index with temperature due to structural rearrangements. On the other hand, the refractive index of the matrix polymer remains nearly constant with temperature. By using a pair of matrix polymer and additive, the refractive indices are equal at low temperatures, and a thermotropic casting resin switching from transparent to light scattering on heating is obtained. However, attempts for commercial production of T-Opal^®^ by Okalux have been deferred.

In 2008, scientific investigations on phase transition systems with adequate switching were published for the first time [12]. Several thermotropic films were prepared by embedding a thermotropic additive in UV cured resin. However, only moderate switching ranges of the normal-hemispherical solar transmittance (ΔT_nh_ < 9%) were reported. Further optimization led to an improved performance and material types with switching ranges of up to 16% [13]. The papers were completed by a definition of the property and performance requirements for thermotropic layers in order to prevent overheating in an all polymeric flat-plate collector [14].

**Figure 2:**
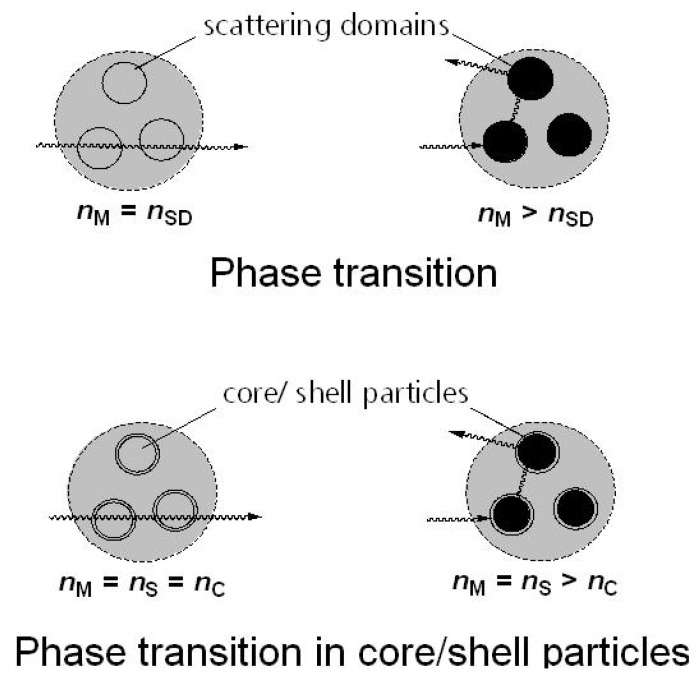
Approaches for thermotropic switching by phase transitions.

Further development applying thermotropic additives as core/shell particles offers several advantages (Figure 2) [15].

All added particles contribute to the thermotropic behavior and, therewith, the amount can be reduced in comparison to application of unprotected domains. Furthermore, the size of the particles and their distribution can be adjusted to result in optimized scattering behavior and improved performance of the thermotropic sun protecting assembly. Furthermore, a polar modified shell prevents the diffusion of the core materials. As an overall result, the long-term stability is essentially enhanced. However, the preparation of well adapted core/shell particles poses a challenge. At all temperatures below the switching point, the refractive indices of all involved components (polymer matrix, particle shell, particle core) should be best matched—especially in the visible wavelength region—to obtain a high transmittance in the off-state in a corresponding thermotropic layer (Figure 2). During the switching process, only the refractive index of the thermotropic core changes significantly. Thus, the particle shell and matrix should have the same refractive index over the whole relevant temperature range and can be regarded as one uniform matrix medium. In other words, here scattering occurs predominantly at the core/shell interface. Accordingly, the scattering domain size is given by the core diameter of the particles. Samples containing particles of high median diameter (4,800 nm) primarily scatter in the forward direction. However), a high back scattering (reflection) efficiency is achieved with smaller particles (300–600 nm). Exploiting the appearing refractive index difference of ~0.07 between particle and matrix at elevated temperatures, the largest difference in the normal-hemispherical transmittance can be found with a particle amount of 6% and a median scattering domain diameter of ~380 nm.

In cooperation with an industrial partner the authors prepared a number of large-area glass laminates (up to 2 m^2^) under industrial conditions demonstrating that a cost-efficient production is possible. The commercialization of the products under the name Solardim^®^Eco was announced emphasizing the high potential of the concept of phase transition in protected domains for various fields of sun protection application.

### 2.8. Thermotropic Polyolefine Films

The extrusion of plastics to films and (multiwall) sheets is widely used to manufacture glazing for greenhouses and buildings and for roofs. Because of this economical relevance, it is interesting to combine the extrusion of plastics with the introduction of thermotropic properties to enable new products for sun protection.

First tests were focused on the utilization of polyethylene (PE—Aldrich; M_w_ ~35,000 g/mol; M_n_ ~7,700 g/mol), which can be compounded and extruded at relatively low temperatures (120 °C–160 °C). Thermotropic particles consisting of a core from an n-alkane mixture and a shell based on of vinyl monomers [15,16] were added directly during the film extrusion as well as by previous compounding. The content of the thermotropic particles was varied in a range from 1% to 10%. In spite of compounding and the use of dispersing agent, higher amounts of particles led to films with inhomogeneous distribution of the thermotropic particles.

A typical film thickness of 300 µm was achieved. Here, the PE films exhibited a high normal-normal transmittance in the range of 70% to 85% at room temperature. In addition, PE films with a thickness of ≥500 µm were prepared. Selected films are summarized in Table 2.

The normal-normal transmittance in the visible range of the PE films decreases clearly with increasing temperature (Table 2), indicating that the added thermotropic particles resisted the thermal and mechanical impact of the extrusion process. Depending on the film thickness and the additive, concentration decreases of up to 36% could be achieved. Such good results could be presented in extruded thin films for the first time ever. However, due to the partially crystalline character of PE, very thick films exhibited a relatively low transmittance at room temperature. A 600 µm thick non-modified PE film showed only a normal-normal transmittance of 43% in the visible range at 15 °C.

**Table 2:** PE films with thermotropic additives.

Thermotropic Film	Additive [%]	Thickness [µm]	Tvis _nn_ [%]		ΔTvis _nn_ [%]
			off	on	
TF 13	10	120	58	22	36
TF 11	5	120	64	37	27
TF 39	5	300	18	9.9	8.1
TF 34	4	500	16	7.2	8.8
TF 35	3	300	26	13	13
PE	0	600	43	42	1.0

Therewith, an important precondition for the application of thermotropic PE films for sun glazing should be the optimized plastic extrusion to films with transmittance as high as possible.

**Figure 3:**
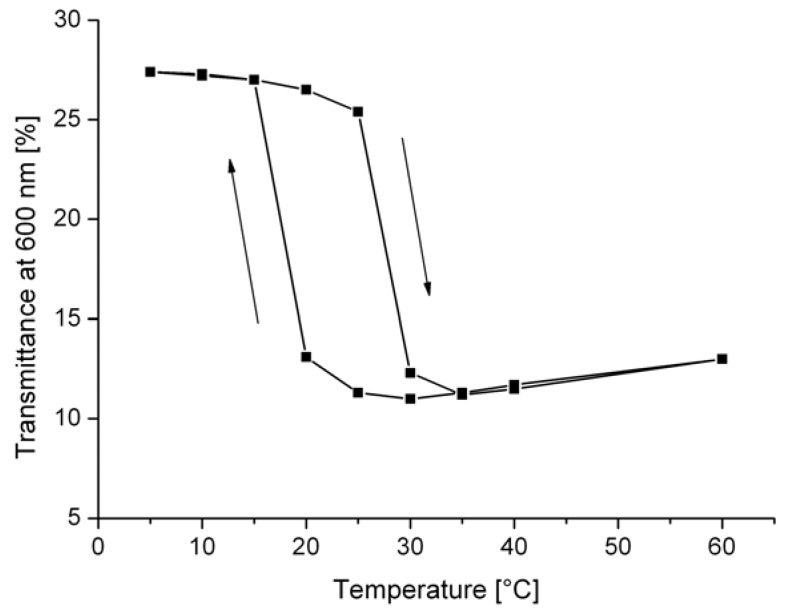
Normal-normal transmittance at 600 nm of the thermotropically modified PE film TF 35 depending on the temperature during heating and cooling [16].

The observed decrease of the transmittance is linked with a thermotropic phase transition and a nonlinear switching could be expected. Figure 3 illustrates this expected change. The switching temperature can be influenced by the composition of the thermotropic additive. However, a super-cooling of about 10 K is often necessary to return to the initial state of the transmittance. The hysteresis between heating and cooling course is also demonstrated in Figure 3.

An increase of the film thickness and the use of dispersing agents during compounding and extrusion led to a further improvement. Temperature-dependent changes of the normal-normal transmittance of >40% could be achieved in these thermotropic PE films.

## 3. Thermochromic Polymer Systems

Thermochromic materials change their visible optical properties in response to temperature. Therewith, the integration of a sensor or actuator functionality into the material itself is enabled. If the absorption of the thermochromic material increases with increasing temperature—sometimes called positive-type or inverse thermochromism—a potential application as smart windows will be possible. Laboratory prototypes already demonstrated this switching effect from a clear, low colored or colorless state into a strong colored state. Furthermore, samples ready for marketing have been presented to the public.

Up to now, the main interest in the field of thermochromic glazing has been focused on inorganic thin layers on glass substrates. Here, the research and development has been reviewed very extensively [59]. The present paper is focused on thermochromic polymer materials. Thermochromism can appear in all different classes of polymers: thermoplastics, duroplastics, coatings or gels [6]. The polymer itself, an embedded additive or a supermolecular system build by the interaction of the polymer with an incorporated additive can cause the thermochromic effect. In the context of sun protection, thermochromic polymer systems obtained by doping the polymer matrix with thermochromic additives are of special interest.

### 3.1. Ligand-Exchange Thermochromic Systems (LETC)

**Figure 4:**
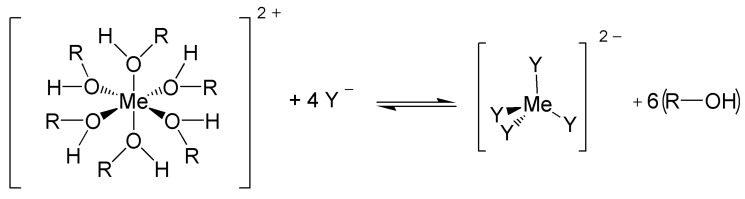
Ligand exchange in Ligand-Exchange Thermochromic Systems (LETC).

For a short time, Pleotint announces Sunlight Responsive Thermochromic (SRT™), a sun protecting glazing based on a thermochromic effect [60]. The development exploits ligand exchange systems integrated in a thin film (Figure 4) [61].

Their thermochromic activity results in a reversible change in absorbance of electromagnetic radiation as the temperature of the system is reversibly changed. The active temperature range of the system is determined by the thermodynamic properties of the LETC reactions. This can include 0 °C to 85 °C for their preferred applications.

**Figure 5:**
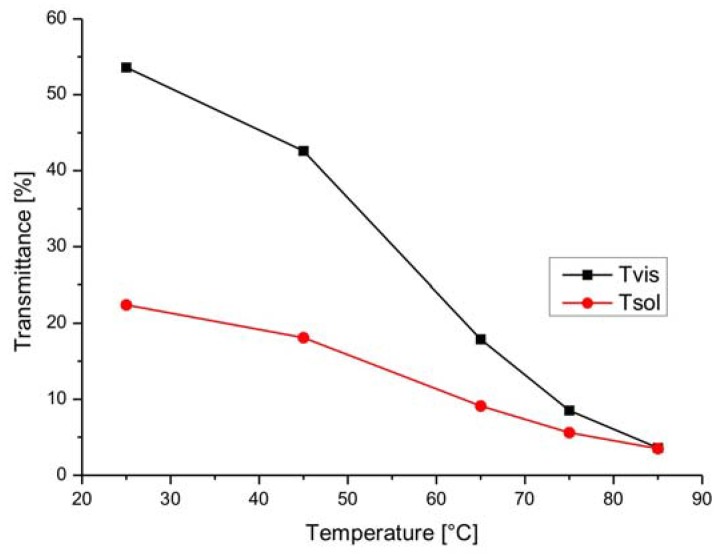
Temperature dependent transmittance behavior of a LETC system (Tvis = Total light transmittance in the visible range 380–770 nm, Tsol = Total solar transmittance range 300–2,500 nm; data from [62]).

The switching behavior of the LETC systems is characterized by a gradual change of the transmittance in the visible as well as in the solar range and by a relatively low transmittance in the off-state at 25 °C (Figure 5). A distinct switching temperature cannot be observed. Energy savings from 17% to 30% are claimed. Information on the long-term stability of the glazing is not yet available.

A further paper emphasizes the importance of this approach [63]. The described thermochromic laminated glazing enables regulation of daylight, automatically adapting dynamically to the continuously changing climatic conditions. Therefore it aids in reducing the energy needs of a building and in providing thermal comfort. Neither electrical power nor a driving unit is required. The polymeric interlayer of the thermochromic laminated glazing is also doped with complexes of transition metals, which change their coordination and transmittance or color of the film under influence of light and heat fluxes. Neutral color conversions from light to dark (grey or brown) and colored ones from rosy or yellow to blue and green were developed.

### 3.2. Leuco Dye-Developer-Solvent Systems

**Figure 6:**
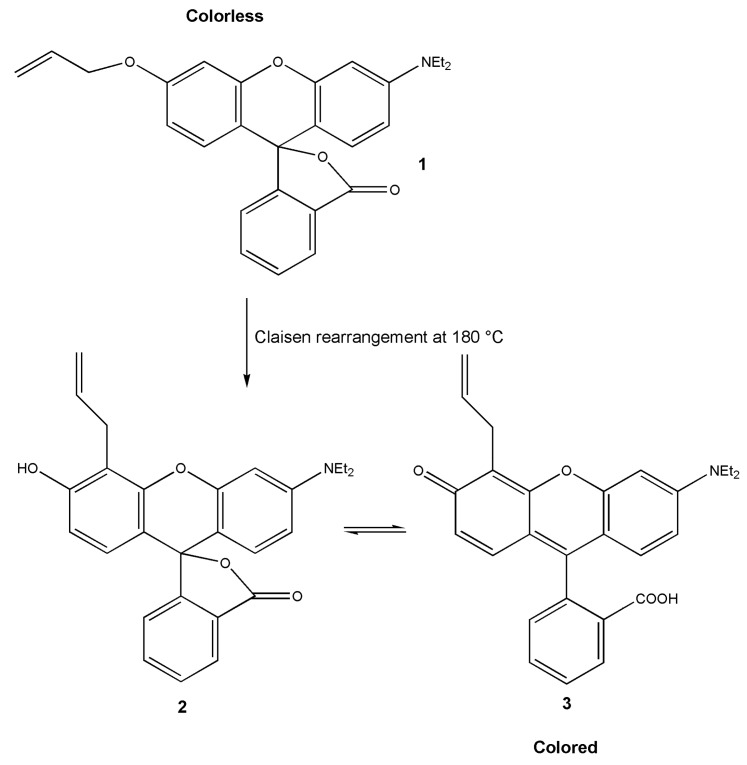
Claisen rearrangement and intramolecular acid-base reaction in a thermoresponsive dye.

The first tests to apply thermo-responsive dyes in thermally activated systems were described in 1992 [64]. The introduced allyl aryl ethers **1** rearrange at 180 °C to a phenol lactone **2**, which in turn undergoes intramolecular proton migration to provide colored **3** (Figure 6).

However, nonsufficient cycle number and missing switching temperatures in a practically useful range prevented its use. Therefore, leuco dye-developer complexes became the most important systems to achieve thermochromic properties for different polymer materials by endowing a separate phase of the thermochromic system in a non-thermochromic polymer matrix. Binary and ternary mixtures are used to enable thermochromic switching from a colorless into a colored state.

To the best of our knowledge, only two examples of this type of thermochromism including two components are described in the literature. In both cases, “amphiphilic” developers with a hydrophobic group (long alkyl chain: C_12_–C_22_) and a rather hydrophilic group are involved [65]. Thus, the thermochromic behavior of a mixture containing a fluoran dye (FD) and octadecylphosphonic acid (ODPA) as the developer were examined. The color-switching temperature of this system is mainly controlled by the melting point of ODPA. When FD/ODPA mixtures were heated to 100 °C, they were completely melted and colored. Slow cooling led to solidification and decolorization initiated at approximately 75 °C. The colorless state caused by slow cooling is formed by fractional crystallization of the developer ODPA (*i.e*., phase separation of dye and developer).

**Figure 7:**
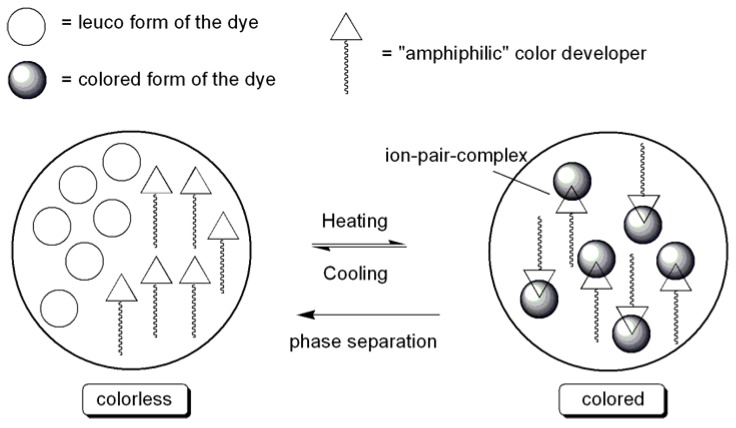
Thermochromic behavior of dye/developer mixtures.

Two-component systems including a leuco dye and a biphenyl developer with a long alkyl chain (4-alkoxy-4’-hydroxybiphenyl) act in a similar way [66]. The mechanism of thermochromic two-component systems leading to a colored state at increased temperature is shown schematically in Figure 7. However, the function of the described binary thermochromic mixtures in polymers has not been tested up to now.

**Figure 8:**
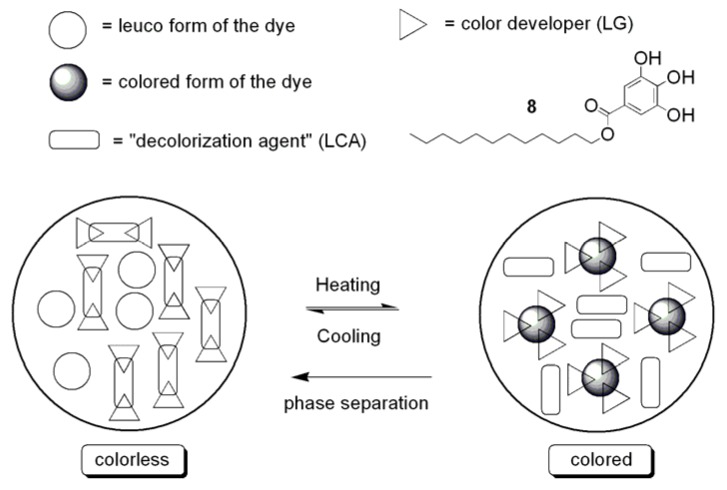
Thermochromic behavior of dye/developer/“decolorizing agent” mixtures.

Thermochromic ternary mixtures consist of a leuco dye (e.g., crystal violet lactone (CVL)), lauryl gallate (LG) as the color developer and a long chain alcohol (LCA, e.g., 1-Tetradecanol) as the solvent (typical molar ratio: CVL/LG/LCA = 1:6:40) [67,68]. A distinguishing feature of these mixtures is a strong attractive interaction between LG and the LCA, leading to the formation of a colorless congruently melting compound of the form (LG)_2_‑LCA. In the molten state, the attractive interaction between LG and the LCA is relatively weak, and the stronger interaction is between LG and CVL, producing a colored complex of the form (LG)_x_-CVL (x = 3–9). However, upon solidification during slow cooling, the color developer LG moves from the colored (LG)_x_-CVL to a colorless (LG)_2_-LCA complex, leading to the decolorization of the mixture. Effectively, the long chain alcohol works not only as the solvent but also as a “decolorization agent” by “disabling the color developer” through complexation. The mechanism of this type of thermochromism including three components is shown schematically in Figure 8.

In further studies, alkyl chain lengths were systematically changed in both the LCA and the alkyl gallate developer [69]. As a result, only LG in combination with 1-tetradecanol showed good results (fast decolorization rate and high color contrast between both states). As the LCA’s alkyl chain is lengthened from C_14_ to C_18_ (developer = LG), the color contrast between both states decreases.

In contrast to the binary mixtures, the ternary positive-type thermochromic mixtures tolerate the presence of a further component like a polymer matrix while maintaining the thermochromic effect. They can be used to prepare plastic films exhibiting the required thermochromic effect and enabling a potential application in sun protection. The first time preparation of thermochromic polyolefines (PE, PP), switching from colorless to colored with increasing temperature, by a relevant technology like film extrusion was the aim of an own development [70]. Extrusion was chosen for the manufacture of the thermochromic polyolefin films, because this technology is capable of future up-scaled production and for commercialization of the thermochromic plastic films.

Investigations showed that the thermochromic composites can successfully be introduced into polymer matrices and that the thermochromic effect is retained. By variation of the composition and of the processing conditions, combinations of leuco dyes, developers, solvents and PE or PP were developed, which exhibit switching temperatures in the range from 60 °C to 80 °C and a change from colorless to colored. 

**Figure 9:**
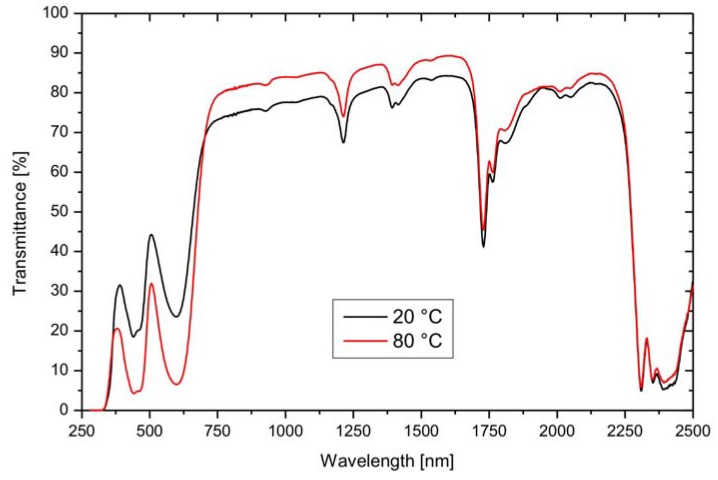
Normal-hemispherical transmittance of a thermochromic film with a green dye at different temperatures [16].

Extruded thermochromic PE films achieved switching ranges of the transmittance of 7–27% in the visible range of the solar radiation. Figure 9 exemplifies the normal-hemispherical transmittance measurements of a thermochromic complex based on a lactone dye.

A considerable decrease of the transmittance with increasing temperature can be observed. The decrease of the transmittance is caused by a strong increase of the absorption and is effective in the visible range of the solar radiation. It is not linked with an increase of the reflection of the incident solar radiation. The molecular switching effect between the different structures cannot contribute to a control of the reflection. A reduction of the transmittance in the visible wavelength range of 27% is only accompanied by a reduction of solar transmittance by 6%.

Comparing the colors of the thermochromic films, it was found that the films forming a blue color with temperature increase are especially suited for sun protection (Table 3). Here, the transmittance decreases from 61% to 34% (ΔT_vis nh_ = 27%). In Table 3, the degrees of the transmittance in the visible and solar wavelength range are summarized for selected thermochromic PE films.

**Table 3:** Degree of visible and solar transmittance of thermochromic PE films.

Dye	Transmittance					
	Tvis_ nh_ [%]		ΔT_vis nh_ [%]	Tsol_ nh _[%]		ΔTsol_ nh_ [%]
	off	on		off	on	
Green	31	15	16	52	47	5
Black	56	49	7	62	56	6
Blue	61	34	27	68	62	6

## 4. Hybrid Thermotropic and Thermochromic Systems 

Polymeric gel networks exhibiting thermotropic properties as well as thermochromic color changes have found considerable interest [71]. The so-called chromogenic materials switch independently from a transparent into a light-scattering state at a distinct temperature and change their color at another temperature. Two strategies have been pursued:

- Introduction of thermochromic properties into thermotropic hydrogels; and
- Addition of a thermotropic character to thermochromic hydrogels.

In a thermotropic hydrogel consisting of polyalkoxide and lithium chloride, water was substituted by a buffer solution and pH-sensitive indicator dyes were then added. The thermotropic behavior of the hydrogel was only slightly influenced by this modification and thermochromism was comparable to thermochromic PVA/borax/surfactant gel networks [72]. The color change was probably raised by the temperature dependent pH-changes of the hydrogel. However, in contrast to the behavior of the phenolic indicator dyes in PVA/borax/surfactant gel networks, the phenol form was stabilized in the polyalkoxide/LiCl hydrogel with increasing temperature. Advantageously, the pH-sensitive phenol phenolate equilibrium can be shifted in both directions with increasing temperature by choosing the appropriate gel network.

**Figure 10:**
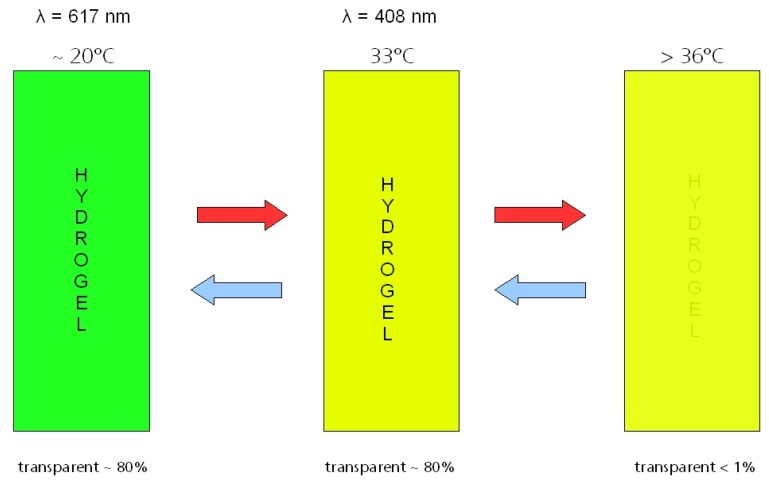
Course of color and transmittance in a system from polyalkoxide/LiCl/bromothymol blue.

An example for the course of color and transmittance in polyalkoxide/LiCl/bromothymol blue hydrogel is given in Figure 10 and demonstrates the introduction of thermochromic properties into thermotropic hydrogels in detail.

The addition of thermotropic properties to a thermochromic hydrogel succeeded by the modification of a PVA/borax/surfactant/phenol red system with small amounts of a polyalkoxide [73]. The employed polyalkoxide exhibits a more hydrophobic behavior than poly(vinyl alcohol). Almost at a concentration of about 0.8% of the polyalkoxide a phase separation process appears at elevated temperature while the color changing process remains unaffected. Independently, with increase of the temperature from 10 °C to 80 °C the color changes step-by-step from yellow to purple. The higher polyalkoxide content leads to an increased rate and a decreased switching temperature of the transmittance change. As a consequence, the normal-normal transmittance decreases from 90% to 20% when the temperature increases from 20 °C to 30 °C.

In contrast, the variation of the concentration of the zwitterionic surfactant affects the thermotropic as well as the thermochromic switching behavior of the gel network. On the one hand, the intensity of the UV/Vis absorption bands appearing at 400 nm and at 563 nm decreases as the surfactant concentration is increased. On the other hand, with increasing surfactant concentration, a slightly increased switching temperature and a weaker switching process are observed for the transparent-opaque transition.

## 5. Nanoparticles in Sun Protection

A new trend in the research and development of sun protection glazing is characterized by the exploitation of nanoparticles embedded in polymer films [74,75] or coated on glass panes [76]. Since nanoparticles can provide spectrally selective absorption without scattering, they can be especially used to dope polymers for use in windows, to provide a clear view while strongly attenuating both solar heat gain and UV, at a lower cost than alternative technologies [77]. The potential for widespread adoption in buildings has clearly been demonstrated, and scopes for further improvements are identified; it is expected that ultimately both cost and performance can be superior.

Further advances enabling improved lighting performance in terms of energy efficient lighting, visual comfort and color management are expected by spectral and angular selective glazing in solar control windows utilizing nanoparticles [78].

Issues affecting the performance of polymers doped with conducting nanoparticles for use with windows are examined in terms of impact on visible and solar transmittance, solar heat gain, and residual scattering [79]. The combination of a visible transmittance fixed in the range of 30–75% with a maximal blocking of the near-infrared component of solar energy in the wavelength range of 750–1,300 nm was emphasized. The absorbance for LaB_6_ and indium tin oxide (ITO) nanoparticles embedded in polymer were studied and found to be quite distinct from each other. But both materials can be used and offer specific advantages and drawbacks. LaB_6_ nanoparticles are very efficient near infrared (NIR) blockers. The absorbance maximum is located near 1000 nm, but unfortunately, overlaps with the visible. In contrast, ITO lies well beyond 1000 nm and is thus far less efficient. Advantageously, it affects the visible only weakly. It was found that ellipsoidal particles are required to explain the properties of the studied LaB_6_ particles, and that scattering can be significant in the NIR while weak in the visible.

These results gave rise to a new approach that considers the nanoparticle size and shape as well as their distribution and orientation in the coating [80]. When nanorods of gold, silver and some other elements are aligned with a preferred orientation with respect to light, their optical extinction characteristics become dependent on the polarization and angle of incidence of the light. This effect could potentially be exploited to produce a “color-change coating”. Surprisingly, it was found that the suspected particle-particle interactions are attenuated if they are between rods of differing aspect ratios. This offered a useful new means of control of the optical properties of coatings of nanorods.

Furthermore, a so-called “solar-thermochromism” using nickel nanoparticles is proposed for an application in sun protection glazing [81]. Translucent composite films of poly(vinylidene fluoride), ionic liquid, and nickel complexes were fabricated using thermal modulation of dissolution, casting, and drying. These films exhibited reversible thermochromic responses at temperatures achievable under sunlight and were evaluated as promising intelligent windows for controlling solar heat entering the built environment.

Further thermochromic films based on silver/polystyrene nanocomposites were prepared by thermal annealing of silver dodecylmercaptide/polystyrene blends at *ca.* 200 °C [82]. The rapid and reversible color switch (from dark-brown to light-yellow) observed at *ca.* 80 °C is probably related to a change in the surface-plasmon absorption of silver nanoparticles that follows variation of inter-particle distances due to polymer expansion. The claimed potential use of this polymeric material for a number of functional applications like optical switches seems to be a more academic approach. In particular, the mentioned preparation of the blends with extrusion could not be reproduced.

## 6. Conclusions

The former and ongoing investigations on the application of thermochromic and thermotropic polymer materials for switchable sun protection glazing were reviewed. New developments setting the future trends in the field of the so-called “smart windows” were examined. 

Hydrogel systems can act as thermochromic as well as thermotropic systems. Their thermochromism is caused by temperature induced matrix effects on the structure of incorporated pH-indicator dyes. From a physical-chemical point of view, an excellent transmittance and the possibility to realize a switching from a colorless into a colored state with increasing temperature are material properties that indicate the outstanding suitability for sun protection glazing. Thermotropic hydrogels based on a temperature initiated phase separation/phase transition process combine an excellent transmittance in the off-state with an outstanding sun protection performance in the on-state. Furthermore, thermochromic and thermotropic effects can be combined in one material system. Also, smart window systems offering passive and active switching can be achieved by an additional outer heating. Nevertheless, in spite of intensive research and ongoing attempts at commercialization, hydrogel based smart windows did not penetrate into the market up until now. From our point of view, in particular, a high manufacturing effort and problems to seal the double glazing prevented the offer of a technologically and economically satisfying solution up until now.

A comparable situation arose with the marketing of phase separating polymer blends. Due to technological problems, the development of smart windows and polymer films based on phase separating polymer blends was interrupted and no products were commercialized. However, promising results were achieved with blends of permanently immiscible polymers with strongly differing temperature dependent behavior of their refractive indices. Such results open up new vistas in the application of polymer blends easily processable by conventional technologies like extrusion for switchable sun protection glazing.

Certainly, extruded thermotropic and thermochromic films will be applied for sun protection in the next couple of years.

Very recently, smart windows based on casting resins doped with thermotropic additives were offered by the TILSE FORMGLAS GmbH [83] in Germany. The products exhibit outstanding long-term stability and excellent switching behavior, providing a satisfying solution for sun protection glazing in glass roofs, glass façade elements, green houses, winter gardens, *etc*.

Moreover, the concept of doping plastics with thermotropic additives of sufficient thermal and mechanical stability will enable passively switching agricultural films and switchable sun protection films for the supplementary fitting of conventional windows. Here, manufacturing is easy through use of conventional technologies like film extrusion ensuring a cost-efficient production of these innovative products. 

The leuco dye-developer-solvent systems enabling thermochromic smart windows have reached a promising development state. A variety of polymer matrices can be doped without deterioration of the mechanical properties of the resulting thermochromic materials. Future research and development activities are focused on the increase of the switching contrast and on the improvement of the light and UV stability; unsatisfying up to now. Here, the colorimetric properties of the leuco dye-based thermochromic materials have to factor in the further development [84]. The color of the thermochromic materials is dependent on the temperature as well as on the thermal history giving rise to color hysteresis.

Ligand-Exchange Thermochromic Systems (LETC) announced by Pleotint [60] (Sunlight Responsive Thermochromic—SRT™) are still in the research and development stage. Their hope to have full-functional windows by October 2009 was not fulfilled, but very recently, they set up an line to produce their film and started accepting orders with the first films coming off the line in September 2010.

Nevertheless, the reviewed actual status of the development in sun protection by use of thermotropic and organic thermochromic materials demonstrates the change of the strategy from ordinary shadow systems to intrinsic solar energy reflection materials based on phase transition components and some promising approaches for their realization on the market are under work. These approaches are complemented by the interesting application of nanoparticles and by totally new concepts in the field of sun protection glazing. Some of these new concepts for switchable material systems will be reports here.

A new chromogenic material was prepared by incorporating a cyano-substituted oligo(*p*-phenylene vinylene) dye into a polyamide by quenching the blend from the melt [85]. The self-assembly of the chromogenic dyes led to pronounced and irreversible changes of the material absorption and fluorescence color by the exposure with outer stimuli. An extension of the concept to reversible changes of the absorption will enable useful effects for an innovative sun protection glazing.

Large-scale shear-ordered photonic crystals have been shown to exhibit unusual thermochromic properties [86]. By balancing the refractive index of the polymer core and composite shell components at room temperature, transparent films are created, which become colored on heating to 150 °C. Since this scattering-based structural color depends only on resonant Bragg scattering, it can be tuned to any wavelength. Since the components are not pigmented, they exhibit an excellent light stability, promoting these systems for an application in sun protection if a decrease to practically relevant switching temperatures can be achieved. Probably, the development of novel piezochromic materials on the basis of selective reflection will also be suitable for application in light valves. To trigger the reversible piezochromic effect, existing pressure changes in bar-range, e.g., between 0.4 bar and 0.9 bar, are sufficient [87]. The aim should be to transfer the trigger signal from pressure to temperature. First examples exist.

In summary, it can be concluded that further developments in the field of the application of thermotropic and thermochromic materials in sun protection glazing keep amazing. This mirrors the effort of the material science to contribute to improved energy efficiency in our normal course of life.
